# CD4/CD8 ratio is associated with structural reorganization of vaccine-induced immune responses in people living with HIV

**DOI:** 10.3389/fimmu.2026.1821444

**Published:** 2026-06-01

**Authors:** Faith Jessica Paran, Mizue Saita, Rieko Oyama, Namiko Nomura, Kaori Saito, Abdullah Khasawneh, Pei Gao, Songling Li, Hendra Saputra Ismanto, Noriyo Nagata, Naoko Iwata, Yasushi Okazaki, Tadaki Suzuki, Yuki Uehara, Kazuhisa Takahashi, Daron M Standley, Yoko Tabe, Toshio Naito

**Affiliations:** 1 Diagnostics and Therapeutics of Intractable Diseases, Intractable Disease Research Center, Graduate School of Medicine, Juntendo University, Bunkyō, Japan; 2 Department of General Medicine, Graduate School of Medicine, Juntendo University, Bunkyō, Japan; 3 Bioresource Research Center, Graduate School of Medicine, Juntendo University, Bunkyō, Japan; 4 Leading Center for the Development and Research of Cancer Medicine, Graduate School of Medicine, Juntendo University, Bunkyō, Japan; 5 Faculty of Pharmacy, Juntendo University Graduate School of Medicine, Bunkyō, Japan; 6 Department of Genome Informatics, Research Institute for Microbial Diseases, Osaka University, Suita, Japan; 7 Department of Infectious Disease Pathology, National Institute of Infectious Diseases, Japan Institute for Health Security, Toyama, Japan; 8 Department of Infectious Disease Pathobiology, Graduate School of Medicine, Chiba University, Chiba, Japan; 9 Department of Clinical Laboratory Medicine, Graduate School of Medicine, Juntendo University, Bunkyō, Japan; 10 Department of Respiratory Medicine, Graduate School of Medicine, Juntendo University, Bunkyō, Japan

**Keywords:** COVID-19 mRNA vaccination, HIV, immune dysregulation, protein-protein-interaction networks, repertoire analysis, scRNA sequencing analysis

## Abstract

**Background:**

Despite effective antiretroviral therapy and sustained viral suppression, people living with HIV (PLWH) continue to exhibit persistent immune activation and low-grade inflammation. Although the CD4/CD8 ratio is routinely monitored as a marker of immune health, the mechanisms underlying residual immune dysregulation remain incompletely understood.

**Methods:**

Peripheral blood mononuclear cells from PLWH and HC were collected before and one month after two doses of mRNA vaccination and analyzed by single-cell RNA sequencing with paired TCR profiling. Differential gene expression (DGE) and protein–protein interaction (PPI) network analyses were performed, and node degree distributions were compared among groups. TCR and bulk BCR repertoires were analyzed, and neutralizing antibody responses were assessed. PLWH were stratified by CD4/CD8 ratio.

**Results:**

Stratification by CD4/CD8 ratio revealed distinct immune states in PLWH. Differential genes from the normal CD4/CD8 ratios showed focused transcriptional activation with coordinated chemokine-associated communication, whereas those with low CD4/CD8 ratios exhibited broad transcriptional dysregulation and structural reorganization of gene-expression networks. This network signature was characterized by altered PPI topology and significantly increased monocyte-specific node degree, independent of overall transcriptional complexity. Repertoire analyses demonstrated clonal skewing and reduced clonotype diversity in the low CD4/CD8 ratio group, consistent with impaired immune coordination.

**Conclusion:**

Vaccine-induced immune responses in PLWH are shaped by network-level organization associated with CD4/CD8 ratio–defined immune states. Low CD4/CD8 ratio group showed increased monocyte-specific PPI connectivity and clonal imbalance, suggesting altered vaccine-induced response dynamics not captured by conventional clinical markers.

## 1 Introduction

Despite effective antiretroviral therapy (ART) achieving durable viral suppression, people living with HIV (PLWH) experience persistent immune activation and dysregulated inflammation, characterized by immune exhaustion, and an increased risk of non-AIDS comorbidities (1–3). In clinical practice, CD4^+^ T-cell counts and the CD4/CD8 ratio are widely used as surrogate markers of immune recovery in PLWH, and a persistently low CD4/CD8 ratio has been linked to adverse clinical outcomes (4). Although clinically informative, these metrics provide only a coarse representation of immune status and offer limited insight into the cellular and molecular organization underlying immune dysfunction.

The global rollout of COVID-19 mRNA vaccines has substantially reduced severe disease and mortality (5). While many PLWH mount measurable antibody responses following vaccination (6), vaccine responsiveness varies widely and is influenced by baseline immune competence. Individuals with low CD4 counts or inverted CD4/CD8 ratios often demonstrate diminished immunogenicity (7). Importantly, protective immunity is determined not only by antibody titers, but also by the interplay of cellular and humoral responses, clonal expansion, and intercellular signaling multifaceted processes that represent system-level immune coordination and cannot be fully captured by conventional clinical metrics alone.

Immune dysfunction in PLWH is increasingly viewed as a failure of coordinated immune network organization rather than isolated changes in cell populations or single pathways (8, 9). However, vaccine response studies in PLWH have largely focused on gene-level or compositional changes, with limited characterization of higher-order immune network structure (10).

Single-cell transcriptomic profiling, often integrated with T-cell and B-cell receptor sequencing, provides high-resolution insight into transcriptional states, clonal dynamics, and intercellular signaling. Applied in a longitudinal vaccination setting, this approach enables assessment of how baseline immune features influence coordinated immune activation following vaccination.

Here, we performed integrated single-cell transcriptomic and immune repertoire analyses of peripheral blood mononuclear cells collected from PLWH and Healthy Controls (HC) before and after COVID-19 vaccination. Stratifying participants by CD4/CD8 ratio, we examined differences in vaccine-induced transcriptional programs, clonal expansion, and intercellular communication networks. By combining single-cell transcriptional profiling with network-based analyses of coordinated molecular interactions, we characterize system-level immune states that extend beyond conventional clinical markers and reveal how baseline immune balance shapes vaccine responsiveness in PLWH.

## 2 Materials and methods

### 2.1 Study participants

In this study, we included eight people living with HIV (PLWH) receiving outpatient care and maintained on stable antiretroviral therapy (ART), along with two HC serving as a comparison group. The ages of the PLWH participants ranged from 29 to 49 years, and the two HCs were 38 and 47 years old. Of the ten participants, nine were male and one was female (**Table 1**). Among the PLWH, four received the BNT162b2 (Pfizer–BioNTech) vaccine and four received mRNA-1273 (Moderna). Among the HCs, one received BNT162b2 and the other received mRNA-1273. All individuals completed a two-dose primary vaccination schedule.

**Table 1:** Demographic and clinical characteristics of the study.

Participant ID	Group*	Gender	Age at vaccination	Vaccine type	CD4/CD8 ratio	
					*Pre*	*Post*
Pt1	Normal	M	46	mRNA-1273	1.8	1.8
Pt2	Normal	F	42	mRNA-1273	2.6	2.2
Pt3	Normal	M	42	BNT162b2	1.8	0.4
Pt4	Normal	M	29	BNT162b2	1.1	1.1
Pt5	Low	M	37	mRNA-1273	0.7	0.6
Pt6	Low	M	49	mRNA-1273	0.3	0.4
Pt7	Low	M	45	BNT162b2	0.1	0.2
Pt8	Low	M	47	BNT162b2	0.9	1.0
HC1	Healthy Control	M	47	mRNA-1273	NA	NA
HC2	Healthy Control	M	38	mRNA-1273	NA	NA

*low: CD4/CD8 ratio less than 1; normal: CD4/CD8 ratio greater than 1 at baseline.

Immune cell subset proportions were obtained from baseline immunophenotyping performed as part of clinical evaluation.

Within the PLWH group, four individuals had a CD4/CD8 ratio within the normal range (≥1; “normal”), whereas the remaining four had a decreased CD4/CD8 ratio (<1; “low”), reflecting heterogeneity in immune status despite successful ART.

All HC and PLWH donors tested negative for SARS-CoV-2 infection both before and after vaccination. Antibodies against the SARS-CoV-2 nucleocapsid (N) protein were assessed as an indicator of prior infection, as mRNA vaccines predominantly elicit responses against the spike (S) protein. Thus, anti-N antibody positivity reflects natural infection rather than vaccination (11).

In addition, serological testing showed that all donors were positive for cytomegalovirus (CMV)–specific IgG prior to SARS-CoV-2 vaccination, consistent with the high prevalence of CMV exposure in adult populations (12). Anti–SARS-CoV-2 N IgG and anti-CMV IgG levels were measured using a commercial assay (SRL Inc., Japan), and seropositivity was defined according to the manufacturer’s cutoff values (Anti-CMV IgG > 6.0; Anti–SARS-CoV-2 N IgG ≥5.0 AU/mL).

This study was approved by the Institutional Review Board (IRB) of Juntendo University Hospital (IRB #21-0244). All participants provided written informed consent. Clinical and demographic characteristics are summarized in **Table 1**, and detailed clinical and immunological parameters are provided in **Supplementary Table 1**.

### 2.2 PBMC sample and serum preparation

Peripheral blood mononuclear cells (PBMCs) were collected from PLWH and HC at two time points: before primary vaccination (pre) and after completion of the two-dose vaccination series (post). At each time point, peripheral blood was drawn into two PBMC preparation tubes (BD Vacutainer CPT™ mononuclear cell preparation tubes, BD Biosciences) and one serum collection tube, according to the manufacturer’s instructions. Isolated PBMCs were washed with phosphate-buffered saline (PBS), resuspended in CELLBANKER 1 Plus (Zenogen Pharma), and cryopreserved at −80 C until use. Serum samples were obtained following centrifugation of the serum collection tubes and stored at −80 C until analysis.

### 2.3 SARS-CoV-2 neutralization assay

Neutralizing antibody titers against the SARS-CoV-2 Wuhan strain were measured as previously described (13). Serum samples were subjected to twofold serial dilutions starting at 1:5 using high-glucose Dulbecco’s Modified Eagle Medium (DMEM; Thermo Scientific) supplemented with 2% fetal bovine serum (Fujifilm Wako Pure Chemicals, Japan). The diluted sera were mixed with SARS-CoV-2 at a viral titer of 6.8 × 10^7^ median tissue culture infectious dose (TCID_50_)/mL in culture supernatant and incubated at 37 C for 1 h. The virus–serum mixtures were then added to VeroE6/TMPRSS2 cells (JCRB1819; JCRB Cell Bank, Japan) seeded in 96-well flat-bottom plates and incubated for 4–6 days at 37 C in a humidified atmosphere containing 5% CO_2_.

After incubation, cells were fixed with 20% formalin (Fujifilm Wako Pure Chemicals) and stained with crystal violet solution (Sigma-Aldrich). Each serum sample was tested in 4–6 replicate wells. Neutralization titers were defined as the reciprocal serum dilution corresponding to ≥50% inhibition of cytopathic effect.

### 2.4 Single-cell RNA library preparation and sequencing

Peripheral blood mononuclear cells (PBMCs) were rapidly thawed at 37 C and resuspended in RPMI-1640 medium (Thermo Scientific) supplemented with 10% (v/v) fetal bovine serum. Only samples with >90% viability were used for downstream processing. Approximately 16,000 cells suspended in phosphate-buffered saline (PBS) containing 0.5% bovine serum albumin (BSA) were loaded onto a Chromium Chip K (10x Genomics), and gel bead-in-emulsion (GEM) generation was performed using Chromium Next GEM Single Cell 5’ GEM Reagent Kits v2 (10x Genomics) and the Chromium Controller (10x Genomics) according to the manufacturer’s instructions.

Transcripts captured within the GEMs were labeled with bead-specific 10x barcodes and unique molecular identifiers (UMIs). Complementary DNA (cDNA) libraries for T cell receptor (TCR) V(D)J sequencing were generated using the Chromium Single Cell V(D)J TCR Amplification Kit (10x Genomics). Gene expression (GEX) and TCR libraries were prepared using the Chromium Single Cell 5’ Library Construction Kit (10x Genomics), Single Index Kit T Set A (10x Genomics), and SI primers (10x Genomics).

The resulting libraries were converted using a Universal Library Conversion Kit (APP-A, MGI). Amplified cDNAs were purified and size-selected using SPRIselect magnetic beads (Beckman Coulter). Library quality and quantity were assessed using the Agilent Bioanalyzer High Sensitivity DNA assay (Agilent) and the KAPA Library Quantification Kit (Kapa Biosystems). Sequencing was performed on the DNBSEQ-G400 platform (MGI) with 100-base paired-end reads. For gene expression libraries, a minimum of 30,000 paired-end reads per cell was consistently achieved, while TCR libraries obtained at least 10,000 paired-end reads per cell.

### 2.5 Single-cell RNA-seq data alignment and V(D)J assembly

Raw sequencing reads were processed using the Cell Ranger software (version 6.1.2; 10x Genomics) using the cellranger multi pipeline. Gene expression (GEX) reads were aligned to the human reference genome GRCh38. V(D)J sequence assembly, alignment, and paired clonotype calling for T cell receptors were performed using the refdata-cellranger-vdj-GRCh38-alts-ensembl-5.0.0 reference (10x Genomics). Complementarity-determining region (CDR) sequences, full-length TCR V(D)J segments, and clonotype frequencies were obtained for downstream analyses.

### 2.6 Single-cell RNA-seq data preprocessing and quality control

Downstream analyses were performed using the R programming language (version 4.2.1) with the Seurat package (version 4.0). All samples were integrated into a single dataset and annotated with donor identity metadata.

Quality control filtering was applied to remove low-quality cells by retaining cells with more than 500 detected transcripts, more than 250 detected genes, and less than 20% mitochondrial gene expression. Genes with zero counts across all cells were excluded from the dataset. After quality filtering, a total of 142,448 cells were retained for downstream analyses, including 28,232 cells from healthy controls and 114,216 cells from PLWH.

### 2.7 Cell-type annotation and group-level comparison of scRNA-seq data

Data were normalized, and highly variable features were identified for dimensionality reduction and clustering. Cell-type annotation was performed using the Azimuth reference mapping approach (14), and uniform manifold approximation and projection (UMAP) was used for visualization of cell clusters. Subsequent analyses were performed at the group level, focusing on overall patterns of cell-type composition and gene expression rather than donor-level variability.

To increase the robustness of reference immune states, healthy control samples were augmented with publicly available scRNA-seq datasets. Processed scRNA-seq gene expression matrices from six HC vaccinated with BNT162b2 (Day 0 and Day 42; GSE171964) were obtained from the Gene Expression Omnibus (GEO) database (15). These datasets included barcodes, features, and count matrices provided by the original authors and were imported directly into R using Seurat.

To assess the comparability between the in-house and publicly available healthy control datasets, we performed integrated analysis and visualized the combined data using UMAP (**Supplementary Figure 1**). Cells from both datasets co-localized within shared clusters without clear separation by dataset origin, indicating comparable transcriptional profiles across major immune cell populations. Although the number of cells in the publicly available dataset was relatively limited, their consistent distribution across established cell-type clusters supports their suitability for inclusion in downstream analyses.

For primary analyses, pre- and post-vaccination cells were pooled across participants within each CD4/CD8 ratio group. Immune states were defined based on CD4/CD8 ratio, and all downstream analyses were conducted on pooled datasets.

Unsupervised clustering was performed on the pooled single-cell dataset to define transcriptionally distinct cell clusters. Cell-type identities were assigned at the cluster level using reference-based annotation and canonical marker gene expression.

Differentially expressed genes (DEGs) were identified by comparing pre- and post-vaccination cells pooled across participants within each group. For each immune cell type, cells were subset, and the identity class was set to vaccination status (pre vs post). DEGs were identified using the Wilcoxon rank-sum test implemented in Seurat (FindAllMarkers), with a fold-change threshold greater than 1.5 and an adjusted p-value < 0.05.

### 2.8 Interferon pathway activity analysis

Interferon signaling activity at baseline was evaluated using module score analysis of single-cell RNA-seq data. Gene sets corresponding to the HALLMARK_INTERFERON_GAMMA_RESPONSE and HALLMARK_INTERFERON_ALPHA_RESPONSE pathways were obtained from the Molecular Signatures Database (MSigDB). Leading-edge genes identified by gene set enrichment analysis (GSEA) were used to construct pathway-specific gene signatures. Module scores were calculated at the single-cell level using the Seurat function AddModuleScore. Analyses were performed on cells pooled across participants within each CD4/CD8 ratio group (healthy control, normal, and low). Statistical comparisons between groups were conducted using the Wilcoxon rank-sum test.

### 2.9 Protein-protein interaction network analysis and visualization

To assess coordinated transcriptional changes at the group level, protein–protein interaction (PPI) network analysis was performed using DEGs. DEGs were identified separately for each immune cell type (CD4+ T cells, CD8+ T cells, B cells, and monocytes) by comparing pre- and post-vaccination cells pooled across participants within each study group (HCs, PLWH with a normal CD4/CD8 ratio, and PLWH with a low CD4/CD8 ratio).This strategy resulted in a total of 12 DEG sets (4 cell types × 3 study groups), each of which was used to construct an independent PPI network.

DEGs were defined using an adjusted p-value < 0.05 and an absolute log2 fold change > 0.3. The resulting gene sets were used as input for the STRING database (version 12.0), with a minimum required interaction score of 0.40 (medium confidence). STRING output tables were exported for downstream analysis.

Protein–protein interaction networks were visualized using Cytoscape (version 3.10.3). Node size was scaled proportionally to degree centrality, while edge width was kept uniform to emphasize overall network topology rather than quantitative interaction strength. Network layouts were generated using default Cytoscape settings, and no clustering algorithms were applied.

### 2.10 T-cell receptor repertoire analysis

Gene expression data matrices obtained from the scRNA-seq analysis were used to identify T cells, which were then integrated with filtered TCR clonotype contigs. Filtered clonotype contigs from the TCR sequence assemblies were merged with the scRNA-seq data for each library using the CombineExpression function in the scRepertoire R package (version 1.7.0) (16). Only cells with TCR sequences, with either an α- or β-, or both TCR chains were included in the repertoire dataset. In subsequent analyses for the TCR, we only investigated the β-chain region. TCR repertoire analysis was performed using the Seurat and scRepertoire packages in R.

### 2.11 TCR clonotype analysis and putative antigen association

To assess clonal expansion within cytotoxic CD8^+^ T cell compartments, we included an analysis of terminally differentiated effector populations. CD8^+^ TEMRA cells are classically defined by cytotoxic function and CD45RA expression; however, CD45 isoforms cannot be reliably distinguished using standard scRNA-seq data due to their regulation by alternative splicing (17).

To approximate CD8^+^ TEMRA cells in the absence of protein-level measurements, we defined a TEMRA-like population based on terminal differentiation and cytotoxicity. A module score was calculated using the Seurat *AddModuleScore* function with the following genes: *PTPRC, KLRG1, GNLY, GZMB, PRF1*, and *FGFBP2* (18). Cells exceeding a defined threshold of the module score were classified as TEMRA-like. These cells were primarily localized within the CD8 TEM cluster (**Supplementary Figure 2**), consistent with the expected transcriptional overlap between effector memory and terminal effector states.

Given that chronic viral infections, particularly CMV co-infection, are known drivers of terminally differentiated CD8^+^ T cell expansion (19), inclusion of this population enabled assessment of highly cytotoxic subsets within the TCR repertoire.

Transcripts encoding the TCR β-chain were recovered from the TCR libraries to characterize clonal dynamics of the repertoire following immunization. Clonotypes were defined based on the complementarity-determining region 3 (CDR3) amino acid sequences of the TCR β-chain using the Cell Ranger analysis pipeline. The β-chain is considered to be more stable than the α-chain and is commonly used for clonotype definition.

Amino acid sequences of the CDR3β region were used to analyze sequence similarities by comparison with publicly available reference databases, including McPAS8 (20), vdjdb (21), TCRex (22), and TCRMatch (23). Predictions were performed according to previously described criteria (24, 25), requiring that CDR3 sequences be of sufficient length and differ by no more than one amino acid from reference sequences. A conservative threshold of 97–100% sequence similarity to reference sequences was applied, and matches meeting this criterion were interpreted as clonotypes with high sequence similarity to previously reported antigen-associated TCRs.

### 2.12 Bulk B-cell receptor sequencing and repertoire analysis

Because BCR sequence recovery from single-cell BCR libraries was insufficient for robust quantitative repertoire analysis, bulk BCR sequencing was performed. Total RNA was isolated from PBMC samples collected before and after vaccination using the RNeasy Plus Mini Kit (Qiagen). Reverse transcription, unique molecular identifier (UMI) barcoding, cDNA amplification, and Illumina adaptor ligation for B-cell heavy and light chain transcripts were performed using the NEBNext Immune Sequencing Kit (Human, New England Biolabs) according to the manufacturer’s instructions. High-throughput paired-end sequencing (2 × 300 bp) was performed on an Illumina NextSeq 2000 platform following the manufacturer’s recommendations. BCR clonotypes were assembled using MiXCR based on V(D)J region sequences (26), employing the built-in preset `neb-human-rna-xcr-umi-nebnext` optimized for the NEBNext Immune Sequencing Kit (Human) BCR. Clonal diversity was assessed using MiXCR outputs, and downstream analyses were performed to evaluate diversity indices, CDR3 length distributions, and V(D)J gene usage.

### 2.13 BCR CDR sequence similarity analysis against reference coronavirus antibodies

Query BCR sequences and reference antibody sequences from the Coronavirus Antibody Database (CoV-AbDab) (27) were aligned and numbered according to the IMGT numbering scheme (27) were annotated and aligned using the IMGT numbering scheme (28). Concatenated CDR1, CDR2, and CDR3 amino acid sequences, referred to as pseudo-sequences, were generated and used to assess sequence similarity between query BCRs and antibodies deposited in the database. To avoid over-representation of duplicated immunoglobulin heavy-chain (IGH) sequences, database records were deduplicated based on identical pseudo-sequences. Sequence similarity between query sequences and reference pseudo-sequences was evaluated by calculating the number of amino acid mismatches (distance) between aligned sequences. A maximum of three mismatches was permitted, and no gaps were allowed.

### 2.14 Statistical analysis

Differentially expressed genes (DEGs) were identified using Wilcoxon rank-sum tests as implemented in the Seurat package. Interferon-related module scores were calculated at the single-cell level and compared between groups within each cell type using Wilcoxon rank-sum tests. (29) Wilcoxon rank-sum tests were also used to compare network connectivity metrics, including node degree distributions, between groups. The Shannon diversity index (30) was used to quantify V gene usage and immunoglobulin isotype diversity in bulk BCR repertoire data. Wilcoxon rank-sum tests were applied to compare clonal diversity indices, isotype diversity indices, and isotype proportions between groups. All statistical tests were two-sided, and p values < 0.05 were considered statistically significant. All statistical analyses were performed using the R programming language.

## 3 Results

### 3.1 Humoral responses following COVID-19 vaccination

Following vaccination, neutralizing antibody titers were induced in all participants, with a decreasing trend observed from HC to PLWH with normal CD4/CD8 ratios and further to those with low CD4/CD8 ratios (**Table 2**; **Supplementary Figure 3**), although no statistically significant differences were detected between groups. Given the variability in humoral responses across participants, we next examined vaccination-induced immune responses at the cellular and transcriptional levels using single-cell RNA sequencing.

**Table 2:** Neutralizing antibody titers against SARS-CoV-2.

Participant ID	Group	Neutralizing antibody titer*	
		*Pre*	*Post*
Pt1	Normal	<5	1:20
Pt2	Normal	<5	1:160
Pt3	Normal	<5	1:10
Pt4	Normal	<5	1:40
Pt5	Low	<5	1:20
Pt6	Low	<5	1:5
Pt7	Low	<5	1:20
Pt8	Low	<5	1:10
HC1	Healthy Control	<5	1:10
HC2	Healthy Control	<5	1:80

*Neutralizing antibodies were used against the Wuhan strain. Titers below the detection limit are shown as <5.

### 3.2 Global immune cell landscape across study groups

Given the variability in humoral responses across participants, we next examined vaccination-induced immune responses at the single-cell transcriptomic level. Single-cell RNA sequencing (scRNA-seq) was performed on a total of 143,250 PBMCs obtained from eight PLWH and eight HCs. The HC dataset comprised two samples generated at Juntendo Hospital and six publicly available datasets retrieved from the GEO database, as described in the Materials and Methods section, all of which were reanalyzed in this study.

**Figure 1:**
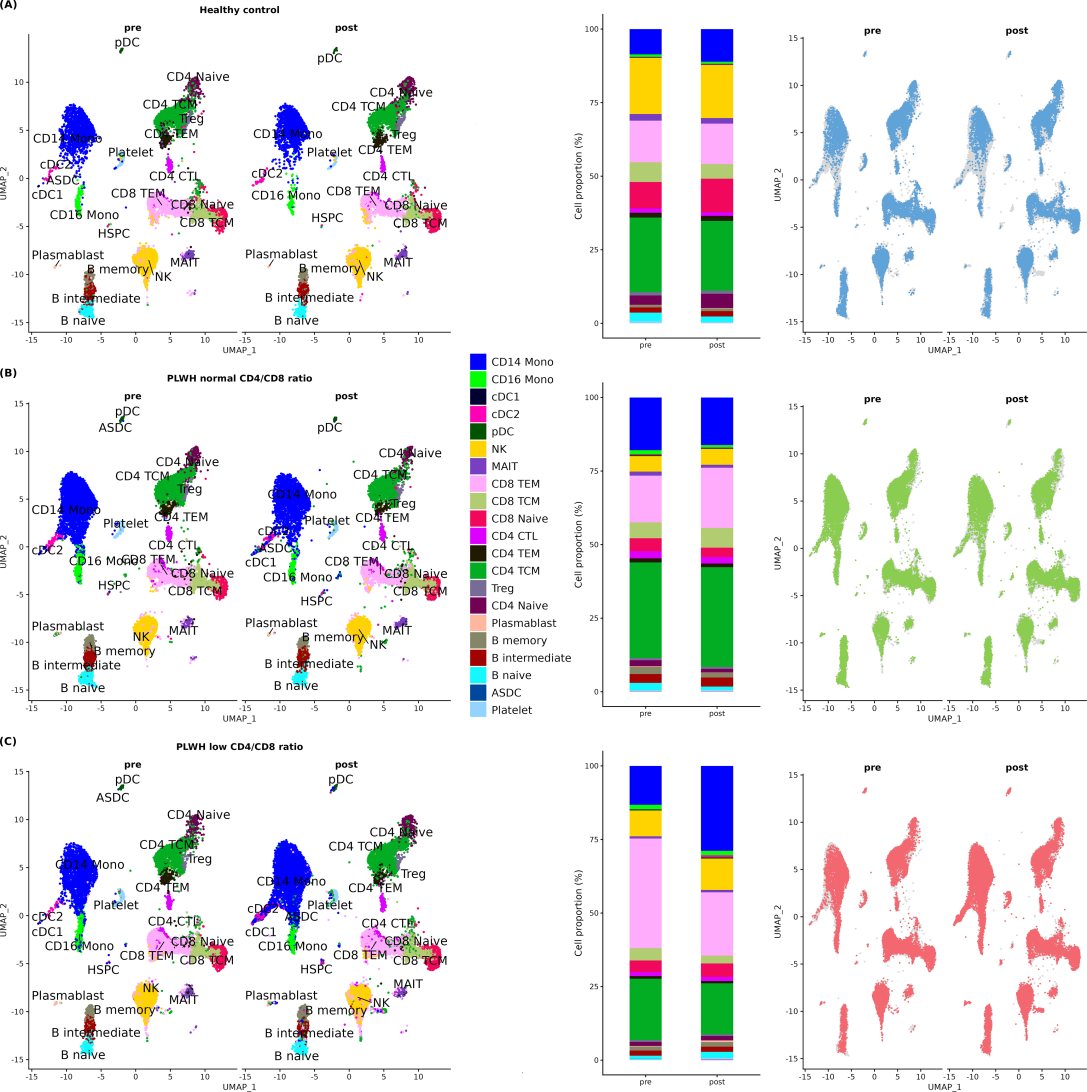
scRNA-seq of peripheral immune cells. UMAP and clustering analysis of peripheral blood mononuclear cells collected from **(a)** healthy control donors, **(b)** PLWH with normal CD4/CD8 ratio, and **(c)** PLWH with low cells CD4/CD8 ratio before and after COVID-19 vaccinations resulted in the identification of 22 transcriptionally distinct clusters. Individual cells are colored by cluster identity determined by Azimuth reference. Bar plots show the proportion of cells. UMAP plots on the third column represent the total number of cells from all donor samples, and individual cells are colored by ratio group (healthy control: blue; PLWH normal CD4/CD8 ratio: green; PLWH with low cells CD4/CD8 ratio: red; all other cells: gray). Mono, monocytes; CD14, CD14-positive; CD16, CD16-positive; CD4, CD4-positive; CD8, CD8-positive; CD4 CTL, CD4-positive cytotoxic T lymphocytes; CD4/CD8 TEM, CD4-/CD8-positive effector memory T cell; CD4/CD8 TCM, CD4-/CD8-positive central memory T cell; Treg, regulatory T cell; MAIT, Mucosal associated invariant T cell; NK, natural killer cell; ASDC, AXL+ dendritic cell; cDC1, CD141-positive myeloid dendritic cell; cDC2, CD1c-positive myeloid dendritic cell; pDC, plasmacytoid dendritic cell.

Using Azimuth-based reference mapping, 22 immune cell populations were consistently annotated across all samples. To obtain an overview of the global immune landscape, cells were pooled within each study group according to vaccination status (pre- and post-vaccination). UMAP visualization of the pooled data revealed broadly conserved immune cell clusters across HCs and PLWH before and after vaccination (**Figure 1**), indicating preservation of overall immune cell architecture.

Participants were analyzed as three groups: HC, PLWH with a normal CD4/CD8 ratio, and PLWH with a low CD4/CD8 ratio. Although major immune cell populations were shared across groups, the normal- and low-ratio PLWH groups exhibited distinct compositional patterns, reflecting differences in baseline immune status. Vaccination was associated with shifts in immune cell proportions in each group (**Figure 1**). Importantly, the direction and magnitude of these changes differed between PLWH subgroups. In particular, the low CD4/CD8 ratio group was characterized at baseline by an expansion of CD8^+^ effector memory T cells (CD8 TEM) and a reduction in CD4^+^ central memory T cells (CD4 TCM) and exhibited a marked increase in CD14^+^ monocytes following vaccination.

Although cells were pooled for group-level analyses, this approach does not capture inter-individual variability. Baseline differences between PLWH subgroups were evident, prompting further analysis of pre-vaccination inflammatory states.

### 3.3 Changes in top differentially expressed genes in PLWH immune cells

**Figure 2:**
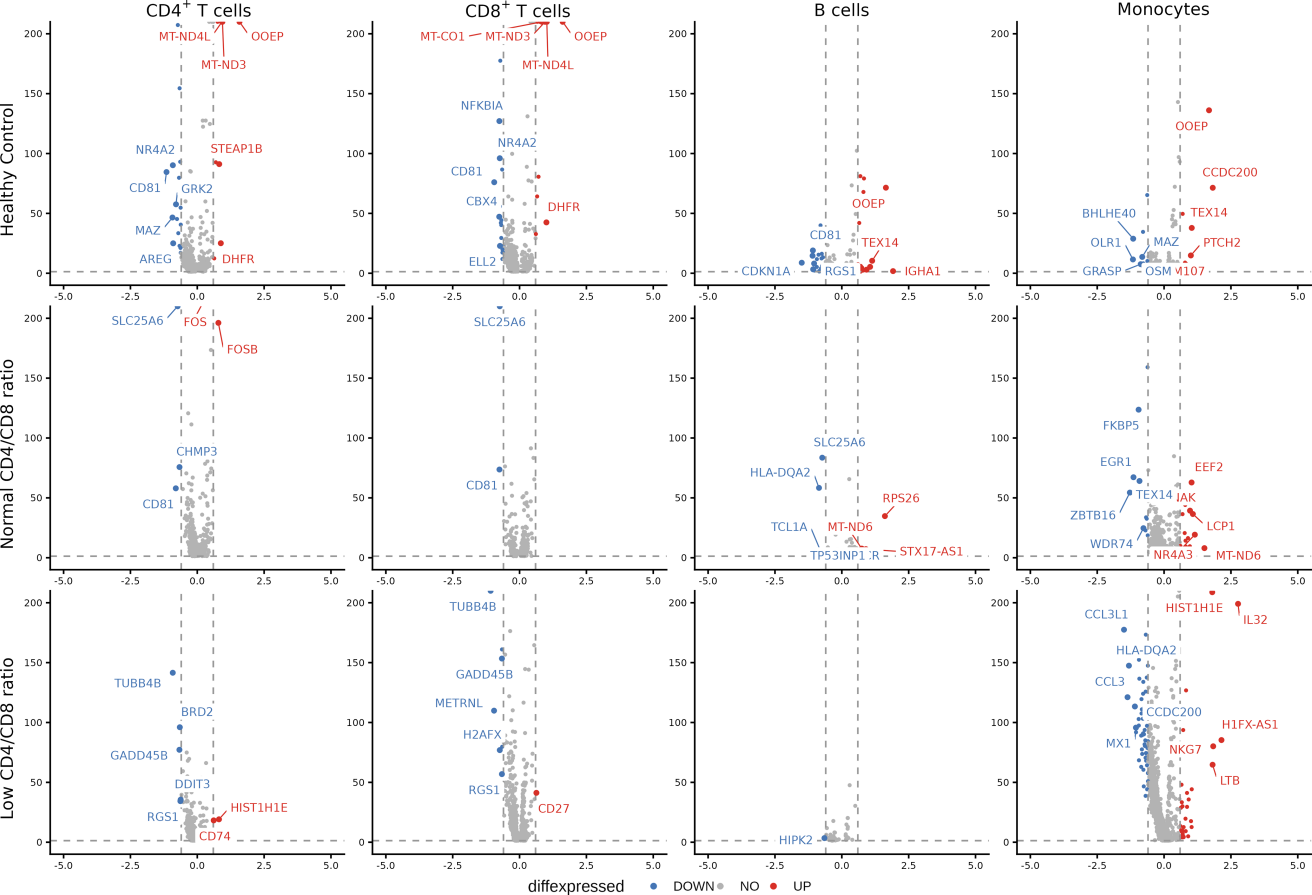
Volcano plot visualization of differential gene expression across immune cell types. Volcano plots showing differentially expressed genes between post- and pre-vaccination samples in HC and PLWH with normal and low CD4/CD8 ratios across major immune cell types (log2FC ≥ 0.5, adjusted p < 0.05, min.pct ≥ 0.25). This plot format integrates both effect size (log2 fold change, x-axis) and statistical significance (−log10 adjusted p-value, y-axis) to enable rapid identification of strongly regulated genes. Red dots indicate significantly upregulated genes, blue dots indicate downregulated genes, and grey dots represent non-significant or low-magnitude changes.

To evaluate immune responses induced by COVID-19 vaccination, we compared gene expression profiles between pre- and post-vaccination samples within each donor group: healthy controls (HCs), PLWH with a normal CD4/CD8 ratio, and PLWH with a low CD4/CD8 ratio. Given baseline differences between groups, analyses focused on within-group vaccination-induced changes rather than direct cross-group comparisons. A complete list of differentially expressed genes (DEGs) is provided in **Supplementary Table 2**, with key genes highlighted in **Figure 2**.

The HC group exhibited partially coordinated transcriptional responses, reflected by the upregulation of overlapping genes after vaccination across CD4 and CD8 T cells, B cells, and monocytes. In contrast, overlapping gene expression patterns were reduced in both PLWH subgroups, suggesting less consistent transcriptional responses across immune cell types compared to HC. In addition, several mitochondrial-related genes displayed cell type–specific expression patterns between HCs and PLWH. For example, *MT-ND4L* was upregulated in CD4 and CD8 T cells in HCs, whereas *MT-ND6* was preferentially upregulated in B cells and monocytes in the PLWH-normal group, highlighting differences in mitochondrial gene expression dynamics between groups.

Cell type–specific patterns were particularly evident within PLWH subgroups. In CD8 T cells, transcriptional changes after vaccination were modest in the normal CD4/CD8 group, whereas the low CD4/CD8 group exhibited an increased number of downregulated genes following vaccination, suggesting a weaker transcriptional response. Similarly, B cells from the low CD4/CD8 group showed fewer differentially expressed genes, with a predominance of downregulation, consistent with reduced transcriptional activation. In contrast, monocytes from the low CD4/CD8 group exhibited a broader spectrum of transcriptional changes, including upregulation of inflammatory and interferon-associated genes such as *IL32*, *MX1*, and *CCL3L1*. In comparison, HCs showed more limited changes in inflammation-associated genes.

To assess whether these group-level transcriptional patterns in monocytes were driven by a subset of individuals, we examined responses at the individual patient level (post- vs pre-vaccination, **Supplementary Figure 4**). As expected, gene-level responses exhibited substantial inter-individual variability, with some individuals showing strong transcriptional changes while others showed relatively modest responses. Multiple individuals within the low CD4/CD8 group contributed to the overall signal, although to varying degrees.

Collectively, these findings indicate that whereas vaccination induces broadly coordinated transcriptional responses across immune cell types in HC, responses in PLWH are more heterogeneous and cell-type restricted. These patterns prompted further investigation into whether the altered genes participate in coordinated interaction networks rather than functioning independently.

### 3.4 Overview of protein–protein interaction networks across immune cell types

**Figure 3:**
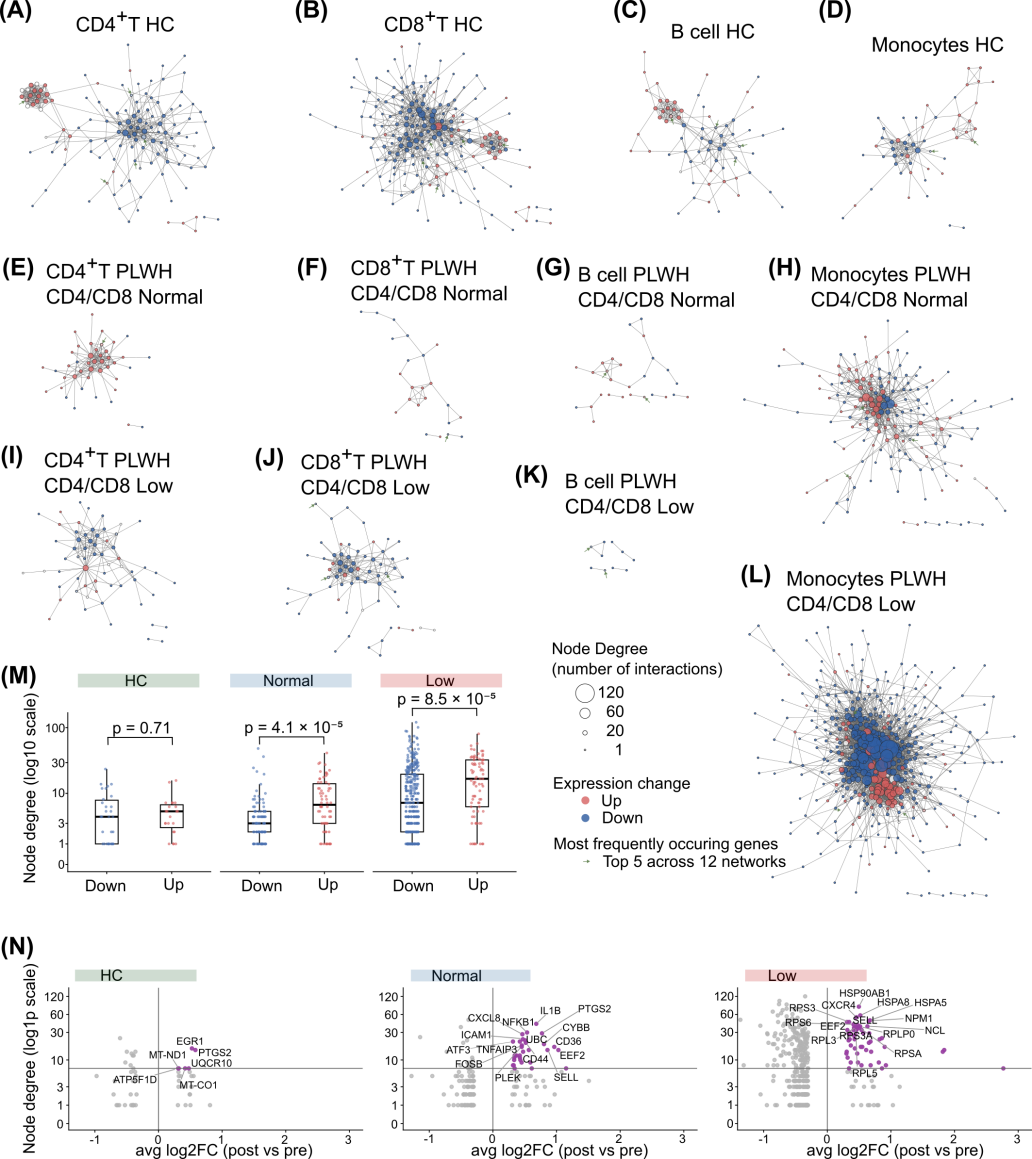
Network reorganization and redistribution of node degree in immune cell populations following COVID-19 vaccination. **(A–L)** Protein–protein interaction (PPI) networks constructed from differentially expressed genes in CD4^+^ T cells, CD8^+^ T cells, B cells, and monocytes from HC (HC), PLWH with a normal CD4/CD8 ratio, and PLWH with a low CD4/CD8 ratio. Nodes represent genes and are colored by direction of expression change following vaccination (red, upregulated; blue, downregulated). Node size is proportional to node degree (number of interactions). The five most frequently occurring genes across the 12 networks are indicated by dark green arrows. **(M)** Distribution of node degree (log10 scale) for upregulated and downregulated genes in monocyte networks across conditions. Each point represents a gene, and boxplots summarize the distribution within each group. Statistical significance was assessed using the Wilcoxon rank sum test. Differences in network node degree were not attributable to differences in monocyte cell abundance or transcriptional complexity (supplementary **Supplementary Figure 2**). **(N)** Scatter plots showing the relationship between average log2 fold change (post vs pre vaccination) and PPI node degree in monocytes across conditions. Dashed vertical lines indicate log2FC = 0, and dashed horizontal lines indicate the node-degree cutoff used to define high-degree nodes. Panels are shown on a linear y-axis; in the low CD4/CD8 group, values above the plotting range were truncated for visualization.

To investigate how vaccination-induced transcriptional changes are organized, we constructed protein–protein interaction (PPI) networks from group-level DEGs (post- vs pre-vaccination) for each immune cell type and study group, generating 12 networks in total. (**Figures 3A–L**).

In CD4^+^ T, CD8^+^ T, and B cells, networks derived from HC were larger and more highly connected than those from PLWH, suggesting reduced coordination of transcriptional responses in lymphocytes from PLWH. In contrast, monocyte networks showed a different pattern (**Figures 3D, H, L**): networks, particularly those with low CD4/CD8 group, exhibited larger and more connected networks compared with controls.

To facilitate comparison across networks, top five shared hub genes were highlighted (**Supplementary Table 3**), and network properties were quantified using node and edge counts (**Supplementary Table 4**). These comparisons indicate cell type–specific differences in network organization, with monocytes showing the most pronounced divergence between PLWH and healthy controls.

### 3.5 Node degree distribution highlights monocyte-specific changes in network centrality

To assess whether these differences reflect changes in network organization, we examined node degree as a measure of gene centrality (**Supplementary Figure 5**). In monocyte-specific networks, upregulated genes (red nodes) in PLWH preferentially occupied high-degree positions, indicating that vaccination-induced genes are centrally integrated within the interaction network. This pattern was not observed in healthy controls.

**Figure 3M** revealed that upregulated genes exhibited significantly higher node degrees than downregulated genes in PLWH monocytes (*p* = 4.1 × 10^-^³ in normal CD4/CD8 PLWH groups and *p* = 8.5 × 10^-5^ in low CD4/CD8 PLWH groups), whereas no difference was observed in controls (*p* = 0.71). In addition, node degree positively correlated with transcriptional upregulation in PLWH (**Figure 3N**), but not in controls.

Inspection of high-degree nodes revealed distinct biological programs (**Figure 3N**). In PLWH with normal CD4/CD8 ratios, centrally positioned upregulated genes included key inflammatory mediators such as *IL1B, NFKB1*, and *CXCL8*, consistent with coordinated immune activation. In contrast, the low CD4/CD8 group showed enrichment of stress response–related genes, particularly heat shock proteins (HSP), among high-degree nodes.

To determine whether these stress-related transcriptional features were present prior to vaccination, we examined differential gene expression in monocytes at the pre-vaccination timepoint (**Supplementary Figure 6**). While several stress response–related genes (e.g., *JUNB, DUSP1, ANXA1*) were upregulated in PLWH compared to HC at baseline, these signatures were not specifically enriched in the low CD4/CD8 group relative to the normal CD4/CD8 group. Instead, similar expression patterns were observed across PLWH subgroups, indicating that the stress-related features observed post-vaccination are not pre-existing characteristics unique to the low CD4/CD8 group but rather emerge following vaccination.

Collectively, these results indicate that, in PLWH monocytes, vaccination responses involve reorganization of network architecture, with induced genes occupying central regulatory positions, rather than simply reflecting differences in expression magnitude.

### 3.6 Antiviral pathway enrichment and intercellular communication differ between PLWH subgroups

**Figure 4:**
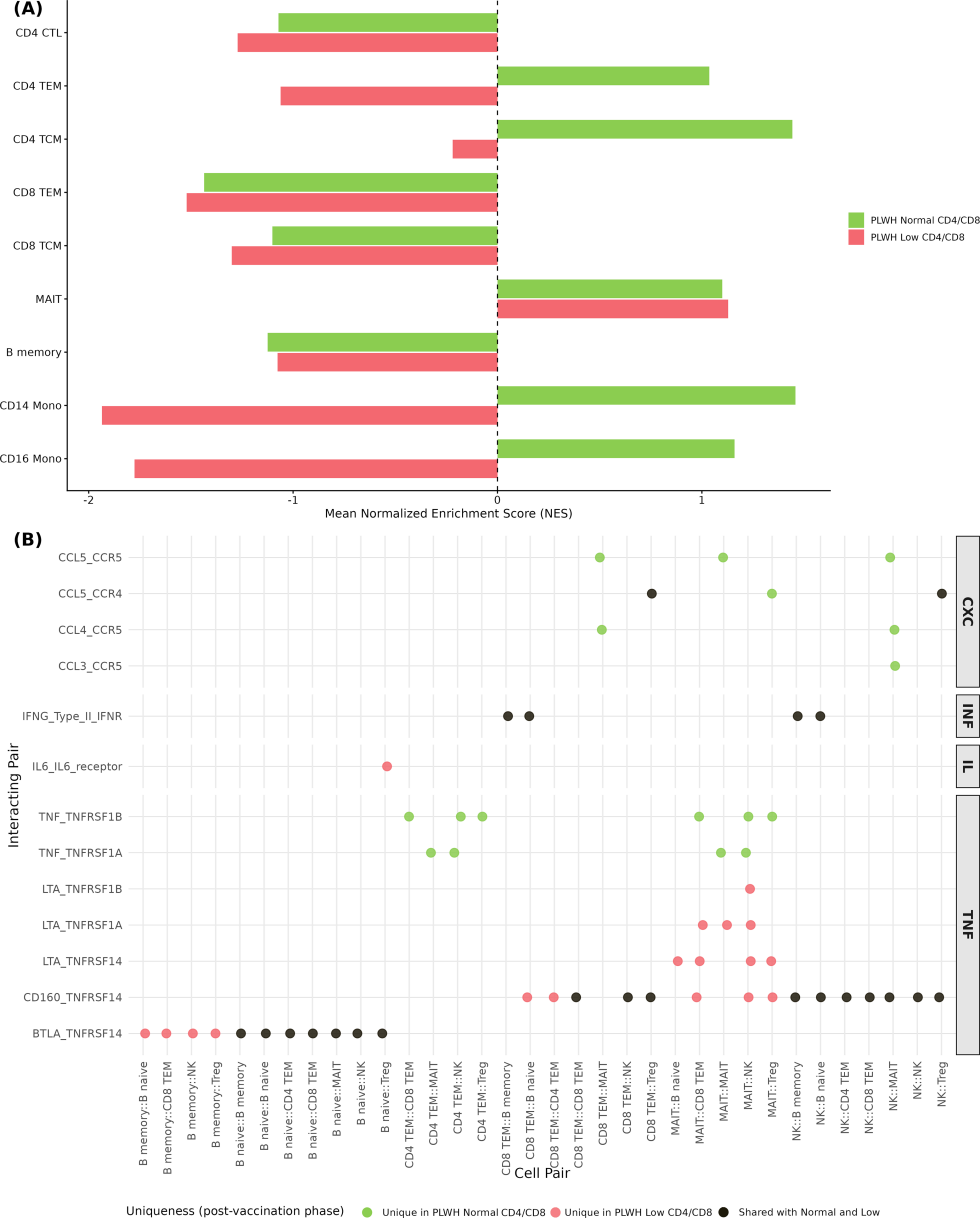
Antiviral pathway enrichment and cytokine-mediated cell–cell interactions following vaccination. **(A)** Gene set enrichment analysis (GSEA) was performed using WilcoxonAUC-ranked genes to compare post- versus pre-vaccination samples across major immune cell subsets. Hallmark pathways associated with antiviral responses (interferon alpha response, interferon gamma response, and TNFα signaling via NFκB) were grouped into the antiviral panel. Bars represent the mean normalized enrichment score (NES) averaged across pathways within the panel for each cell type. Positive NES values indicate enrichment post-vaccination, whereas negative NES values indicate enrichment pre-vaccination. Green bars denote PLWH individuals with normal CD4/CD8 ratios, and red bars denote individuals with low CD4/CD8 ratios. **(B)** Dot plots illustrate ligand–receptor interactions involved in immune signaling pathways during the post-vaccination phase, comparing PLWH individuals with normal and low CD4/CD8 ratios. Interactions are grouped into chemokine (CXC), interferon (IFN), interleukin (IL), and tumor necrosis factor (TNF) signaling categories. The x-axis represents interacting immune cell pairs, and the y-axis indicates ligand–receptor pairs. Dot size reflects the average interaction score, and dot color indicates whether interactions are shared between groups or unique to either the normal or low CD4/CD8 ratio group. Ligand–receptor pairs were identified using CellPhoneDB.

To further interpret subgroup-specific responses in functional terms, we examined antiviral pathway enrichment (Hallmark pathways) and ligand–receptor interactions following vaccination. Antiviral pathway enrichment (post vs pre) differed between PLWH subgroups in a cell type–dependent manner (**Figure 4A**). The low CD4/CD8 group showed stronger enrichment in CD4 TEM/TCM cells and CD14/CD16 monocytes compared with the normal CD4/CD8 group, indicating distinct antiviral transcriptional responses across immune compartments.

Although individual-level analysis revealed substantial variability within the low CD4/CD8 group (**Supplementary Figure 7**), consistent pathway-level trends were observed across multiple patients, indicating that group-level findings were not driven by a small number of outliers.

We next examined intercellular communication using ligand–receptor analysis (**Figure 4B**). Distinct subgroup-specific interaction patterns were observed across major signaling axes, including chemokine, interferon, interleukin, and TNF-related pathways. Chemokine interactions (e.g., *CCL3/CCL4/CCL5* with *CCR5/CCR4*) were preferentially enriched in the normal CD4/CD8 group. In contrast, *TNF*-related signaling differed between subgroups in both ligand usage and cellular context. *TNF–TNFR* interactions were enhanced in CD4 TEM and MAIT cells in the normal CD4/CD8 group, whereas *LTA–TNFR* interactions were selectively increased in MAIT cells in the low CD4/CD8 group. Furthermore, *CD160–TNFR* interactions in CD8 TEM and MAIT cells, as well as *BTLA–TNFR* interactions in B memory cells, were increased in the low CD4/CD8 group. These patterns indicate subgroup-specific differences in post-vaccination communication networks and provide functional context for the network reorganization observed in monocytes, particularly along the *TNF/LTA* signaling axis.

### 3.7 Altered TCR repertoire dynamics in PLWH following vaccination

**Figure 5:**
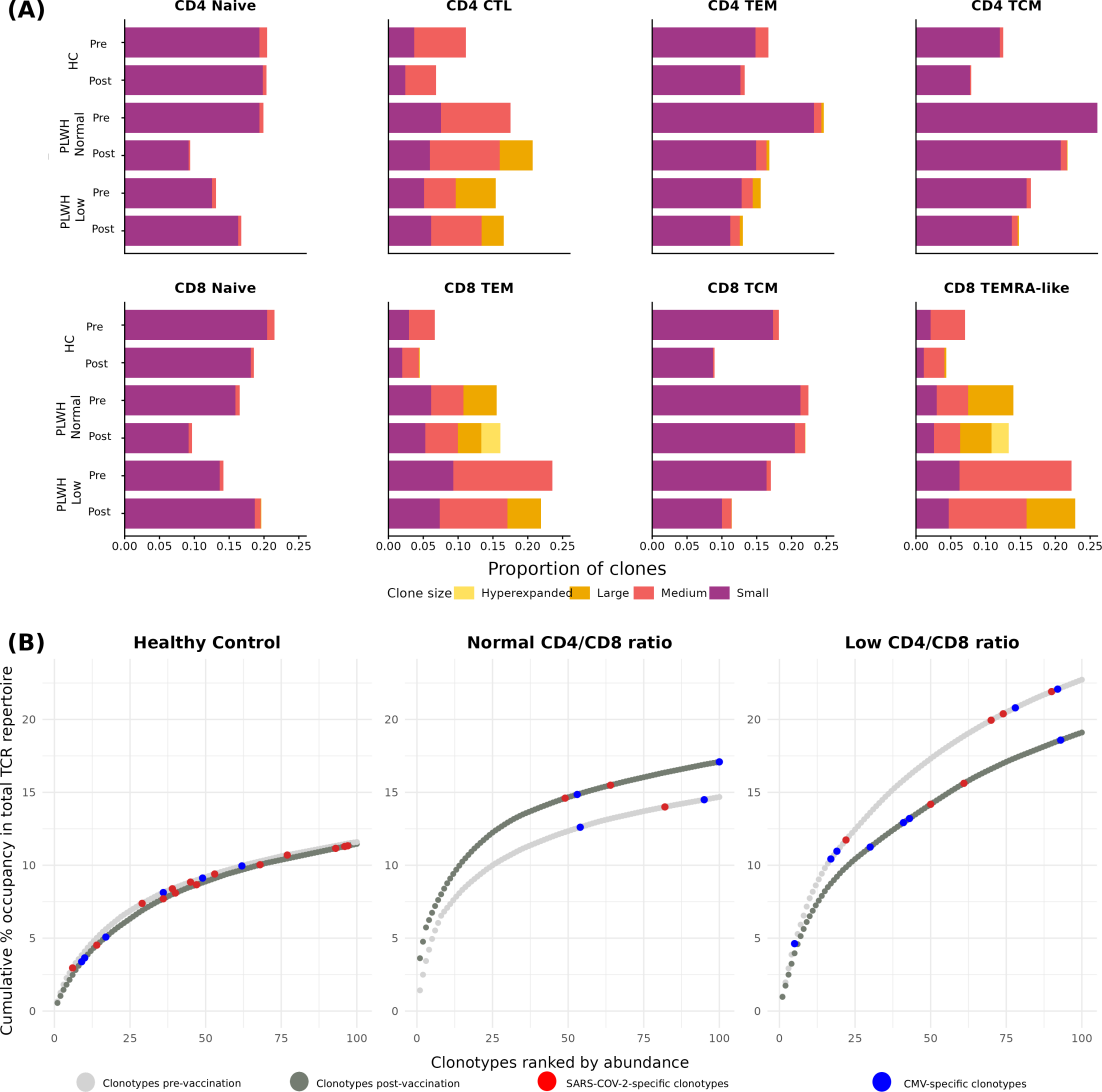
T cell clonal architecture and antigen-associated clonotype expansion following vaccination. **(A)** Clonal occupancy patterns across T cell subsets and cohorts. Clonal occupancy was assessed for major T cell subsets in HC and people living with HIV (PLWH) stratified by CD4/CD8 ratio (normal and low) before and after vaccination. Horizontal stacked bar plots depict the proportion of TCR clonotypes classified into five clone size categories based on their relative abundance within each subset: single (0 < X ≤ 1×10^-4^), small (1×10^-4^ < X ≤ 0.001), medium (0.001 < X ≤ 0.01), large (0.01 < X ≤ 0.1), and hyperexpanded (0.1 < X ≤ 1). For each T cell subset and cohort, clonotypes were grouped into these categories, and the fraction of clonotypes in each category was calculated relative to the total number of clonotypes detected in that subset and cohort. Colors indicate clone size categories. **(B)** Cumulative occupancy of top-ranked TCR clonotypes. Clonotypes were ranked by abundance within each group, and the top 100 clonotypes are shown. The y-axis represents the cumulative percentage of total TCR repertoire occupancy. Green dots indicate clonotypes predicted to be specific for SARS-CoV-2 antigens, and blue dots indicate clonotypes predicted to be specific for cytomegalovirus (CMV) antigens.

Clonal expansion patterns differed between HC and PLWH following vaccination (**Figure 5A**). In HC, large and hyperexpanded clones were not observed across all T cell subsets either before or after vaccination, and no clear trend toward post-vaccination clonal expansion was detected. In contrast, PLWH exhibited a marked increase in expanded clones within the cytotoxic CD4 CTL and CD8 TEM compartments. Specifically, at baseline prior to vaccination, large clones were already present in CD8 TEM cells in the normal CD4/CD8 group and in CD4 CTL cells in the low CD4/CD8 group. Following vaccination, clonal expansion of CD8 TEM cells became pronounced in PLWH, including both the normal and low CD4/CD8 ratio groups, whereas HC exhibited a tendency toward clonal contraction.

**Figure 6:**
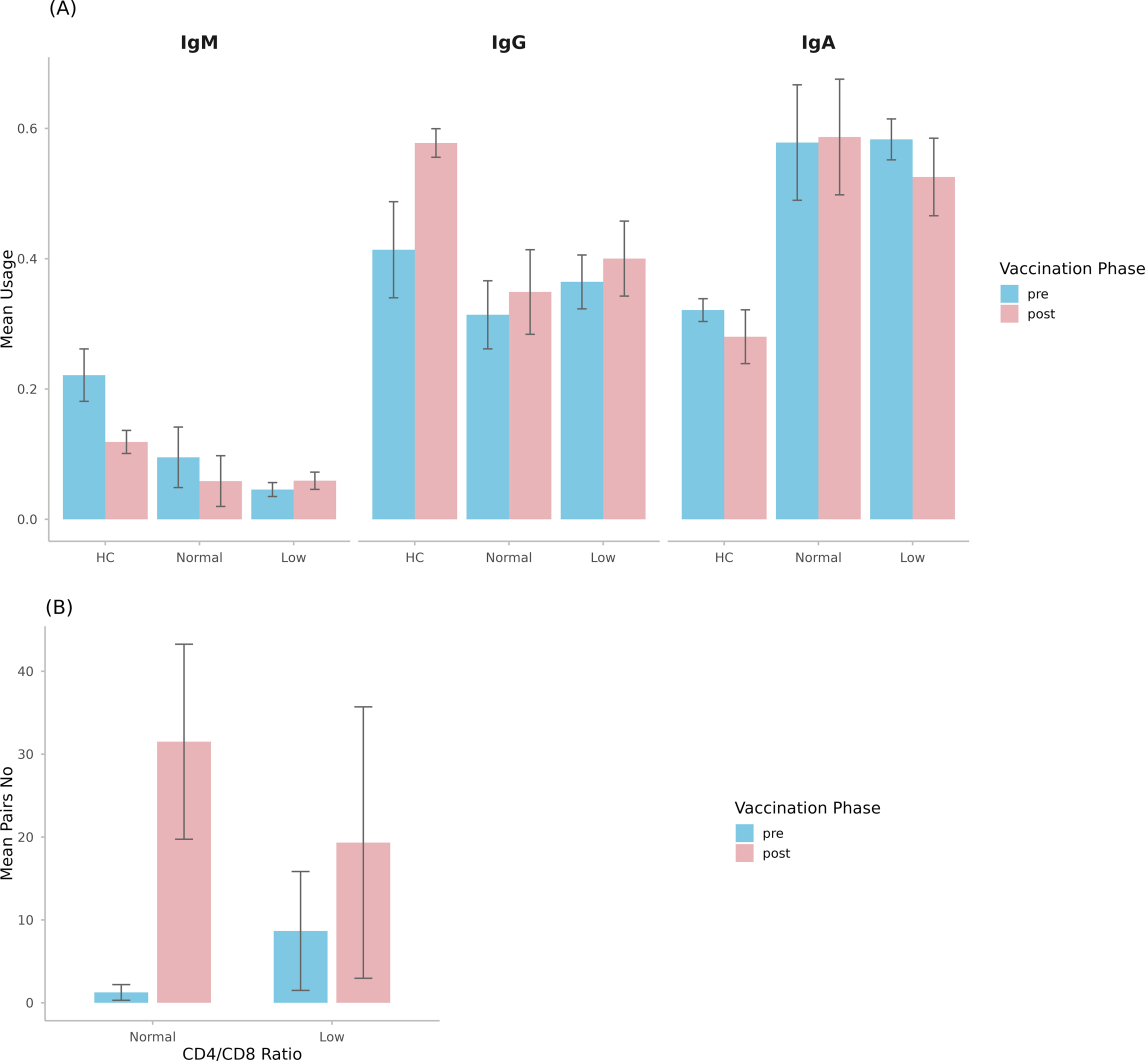
BCR repertoire analysis using bulk RNA sequencing. **(A)** Isotype usage of healthy and PLWH donors before and after COVID-19 vaccinations. **(B)** The number of similar IGH sequence pairs (Pairs No) before and after vaccination matched to known antibodies from the coronavirus antibody database (CoV-AbDab). Bars represent mean values, and error bars indicate SEM.

To assess the contribution of terminally differentiated CD8^+^ T cells, we identified a population of TEMRA-like cells based on transcriptional signatures associated with cytotoxicity and terminal differentiation (see Materials and Methods). Clonal occupancy analysis revealed that TEMRA-like cells exhibited expansion patterns comparable to CD8 TEM cells (**Figure 6A**), reflecting their overlapping transcriptional profiles. These findings suggest that highly cytotoxic, terminally differentiated CD8^+^ T cells contribute to the expanded repertoire in PLWH, although they do not represent a distinct clonally dominant compartment.

Given the high prevalence of CMV co-infection in PLWH and its established role in driving the expansion of terminally differentiated CD8^+^ T cells, we next examined whether known antigen specificities could explain the observed repertoire skewing (**Figure 5B**). Cumulative frequency curves showed that, in both PLWH subgroups, the dominance of top-ranked clonotypes was greater than in HC both before and after vaccination.

However, annotation of TCR sequences against public antigen-specific databases revealed that SARS-CoV-2-associated clonotypes were detected across groups but did not account for the observed clonal expansions (**Supplementary Table 5**). CMV-associated clonotypes were also present in all groups, consistent with widespread CMV seropositivity, but were not considered the primary drivers of the skewing patterns. The majority of clonotypes could not be matched to known antigen specificities. It should be noted that TCR annotation using public databases is limited by incomplete clonotype coverage and does not account for TCR cross-reactivity, which may underestimate antigen-specific responses.

Together, these findings indicate that the expanded TCR repertoire observed in PLWH reflects broad antigen-driven or bystander expansion rather than selective amplification of known SARS-CoV-2-specific clones, potentially shaped by chronic immune activation associated with HIV and CMV co-infection.

### 3.8 BCR repertoire dynamics following vaccination

Finally, to characterize B-cell clonal responses following vaccination in PLWH, bulk RNA sequencing analysis was performed, identifying 18,617 unique BCR clonotypes from a total of 20,011 sequences obtained. As this analysis was conducted at the bulk level, results reflect the overall B cell compartment rather than specific subsets such as plasmablasts or long-lived plasma cells.

As shown in **Figure 6A**, isotype distributions exhibited modest shifts one month after completion of vaccination; however, these changes did not reach statistical significance across groups. Although IgM usage tended to decrease and IgG usage appeared to increase following vaccination in HC, paired comparisons were not statistically significant. In PLWH, IgA usage was elevated at baseline and remained comparatively high post-vaccination, but no significant pre–post differences were detected within either the normal or low CD4/CD8 subgroups. Likewise, comparisons across clinical groups did not reveal significant differences for IgM, IgG, or IgA usage.

These findings suggest that overall isotype distribution remained relatively stable at the bulk repertoire level, potentially reflecting either limited cohort size or the predominance of clonotype-level rather than isotype-level shifts following vaccination.

To further evaluate vaccine-associated specificity, BCR clonotypes were compared with the CoV-AbDab reference database based on CDR3 sequence identity (**Figure 6B**). Individuals with a normal CD4/CD8 ratio demonstrated an increase in sequence pairs matching known SARS-CoV-2 antibodies following vaccination; however, this increase did not reach statistical significance (paired t-test, *p =* 0.081). No significant changes were observed in the low CD4/CD8 ratio group (*p =* 0.652). These data suggest a trend toward preferential expansion of vaccine-associated BCR clonotypes in PLWH with preserved CD4/CD8 ratios, although this effect was modest at the bulk level and did not reach statistical significance in the present cohort.

## 4 Discussion

Despite effective antiretroviral therapy (ART), people living with HIV (PLWH) show reduced vaccine responses to mRNA SARS-CoV-2 vaccines, particularly at lower CD4 counts (4, 31, 32), in the context of persistent immune activation and inflammation (8, 33). Here, single-cell transcriptomic analysis reveals that vaccine responses in PLWH are better captured by coordinated immune network changes than by consistent differential gene expression, with network-based analyses uncovering structured interaction-level remodeling not detected by conventional approaches.

Differences in network organization were most evident in monocytes. In HC, vaccine-responsive networks appeared relatively compact and organized into defined subnetworks distinct from broader immune signaling patterns, consistent with relatively modular network organization. In contrast, PLWH with low CD4/CD8 ratios exhibited expanded and more densely interconnected monocyte-derived protein–protein interaction networks with increased gene centrality. While greater connectivity can reflect activation, excessive interconnection may indicate altered modular organization and dysregulated signaling (34).

Given the central role of monocytes in shaping downstream adaptive immunity, this expanded network architecture in the low CD4/CD8 group may influence T cell priming and immune memory formation (35). These findings suggest that vaccination in PLWH involves not only changes in gene expression but also shifts in monocyte network organization, characterized by increased network connectivity and centrality in individuals with persistent immune imbalance. This expanded network structure observed in the low CD4/CD8 group after COVID-19 vaccination may reflect responses occurring within a pre-existing inflammatory context, potentially shaping the organization of antiviral immune responses.

Intercellular communication highlighted context-dependent differences involving MAIT cells, consistent with the central role of monocytes in orchestrating innate–adaptive crosstalk. In individuals with normal CD4/CD8 ratios, pre-vaccination MAIT interactions were detectable and aligned with chemokine-associated signaling after vaccination, suggesting coordinated immune responses (36, 37). In contrast, in the low CD4/CD8 group, innate-like populations such as MAIT cells appeared to engage alternative pathways to maintain immune communication. Whether this reflects effective compensation or dysregulated coordination cannot be determined from the present data and warrants further investigation.

Intercellular communication analysis further supported divergent modes of cell talk. In the normal CD4/CD8 group, monocyte–cytotoxic cell interactions remained prominent and were enriched for chemokine-associated signaling, paralleling the coordinated intracellular network organization. In contrast, the low CD4/CD8 group demonstrated enrichment of *TNF*-superfamily interactions involving B cells, indicating a shift toward inflammatory regulatory circuits. Prior studies have linked *TNF*- and lymphotoxin-mediated pathways to altered B cell differentiation and germinal center structure during chronic HIV infection (38), and enrichment of *BTLA–TNFRSF14* signaling may reflect attempts to restrain immune activation (39). Together, these findings suggest that normal CD4/CD8 ratio support coordinated immune communication following vaccination, whereas low ratios are associated with a compensatory shift toward inflammation-associated interaction networks.

TCR repertoire analysis provided additional evidence of constrained immune organization in individuals with low CD4/CD8 ratios. Although clonal expansion occurred after vaccination, it was not accompanied by preferential enrichment of SARS-CoV-2–associated clonotypes based on available reference databases. However, given the limited coverage of current TCR annotation resources, this finding may reflect incomplete annotation rather than a true absence of antigen-specific expansion and should be interpreted with caution. This pattern may nevertheless be influenced by converging factors, including chronic immune activation, reduced naïve T cells availability, and the expansion of clonotypes targeting latent viruses such as cytomegalovirus (CMV) (1, 40).

In particular, CMV co-infection, common in both PLWH and healthy individuals, is a well-established driver of terminally differentiated CD8^+^ T cell populations, including TEMRA cells (18). Persistent CMV-specific clonal dominance may limit repertoire flexibility through immune resource competition, reducing the capacity to mount highly specific responses to new antigens (41). These findings suggest that vaccine responsiveness in PLWH is influenced by factors beyond HIV-associated immune deficiency, including chronic co-infections.

Humoral immunity also demonstrated qualitative differences not fully captured by neutralizing antibody titers alone. Although overall antibody levels differed only modestly between PLWH and HC, BCR repertoire analysis revealed persistent IgA dominance and reduced convergence toward SARS-CoV-2–specific clonotypes in the low CD4/CD8 group. This pattern is consistent with impaired germinal center activity and reduced functional CD4 T cell help, which are critical for effective class switching and affinity maturation (42, 43). In contrast, individuals with normal CD4/CD8 ratios exhibited greater convergence toward antigen-specific BCR clonotypes, underscoring the importance of coordinated T–B cell interactions in generating durable humoral immunity. These findings highlight the limitations of relying solely on antibody titers to assess vaccine efficacy in immunocompromised populations.

This study has several limitations. The cohort size was modest and consisted predominantly of Japanese male participants, which may limit generalizability. Although the PLWH cohort included both Pfizer-BioNTech COVID-19 vaccine and Moderna COVID-19 vaccine recipients, analyses were restricted to Pfizer-BioNTech due to sample size constraints and the absence of Moderna-vaccinated healthy controls, limiting assessment of platform-specific effects. In addition, our analyses are observational and lack mechanistic validation, precluding direct conclusions on causality. Epitope assignment based on public databases may underestimate antigen-specific TCR diversity, and PBMC-based profiling does not capture tissue-resident immune responses. The role of MAIT cells in long-term memory also remains unclear.

Despite these limitations, prior studies reporting impaired T cell proliferation (44, 45) and cytokine production (46), in PLWH with low CD4/CD8 ratios support the biological relevance of our findings. Furthermore, the consistent patterns observed across transcriptional, clonal, and intercellular network analyses reinforce the robustness of the results and provide a framework for future mechanistic studies.

In conclusion, CD4/CD8-defined immune states are associated with distinct structural patterns of vaccine-induced coordination in PLWH. Rather than reflecting isolated transcriptional changes, altered vaccine responsiveness appears rooted in reorganization of immune interaction networks, clonal dynamics, and intercellular communication. These findings underscore the importance of assessing immune organization beyond conventional clinical markers and provide a rationale for stratified vaccination strategies that account for baseline immune status in immunocompromised populations.

## Data Availability

The datasets generated and analyzed during the current study have been deposited in the NCBI repositories under BioProject accession number PRJNA1465100 for “Single-cell transcriptomic and immune repertoire profiling of COVID-19 mRNA vaccination responses in PLWH” and are available through the Sequence Read Archive and Gene Expression Omnibus (GEO) under accession number GSE332711.

## Supplementary material

Supplementary Figure 1 Integration of in-house and publicly available healthy control datasets. UMAP visualization of integrated single-cell RNA-seq data from in-house healthy controls and a publicly available healthy control dataset (GSE171964). Cells are colored by dataset origin (In-house HC vs. GSE HC). Cells from both datasets co-localize across shared clusters without evident dataset-specific segregation, indicating comparable transcriptional profiles. Despite the smaller number of cells in the publicly available dataset, its distribution across major immune cell populations supports its inclusion in downstream analyses.

Supplementary Figure 2 Identification and distribution of TEMRA-like CD8^+^ T cells across cohorts and vaccination states. **(A)** UMAP projection of T cells colored by Azimuth-derived cell type annotations, split by cohort and vaccination status. Cell type labels are overlaid for reference. **(B)** UMAP highlighting TEMRA-like cells defined by a cytotoxicity and terminal differentiation gene module (*PTPRC, KLRG1, GNLY, GZMB, PRF1, FGFBP2*), as described in Methods. Cells exceeding the module score threshold were classified as “TEMRA-like,” while remaining CD8 TEM cells and other populations are shown separately. TEMRA-like cells predominantly localize within the CD8 TEM compartment, consistent with the transcriptional continuum between effector memory and terminally differentiated cytotoxic CD8^+^ T cells.

Supplementary Figure 3 Neutralizing antibody (Nab) titers among healthy control, normal CD4/CD8 ratio, and low CD4/CD8 ratio groups after COVID-19 vaccinations. Analysis of Nab titers against the SARS-CoV-2 Wuhan strain for serum samples from 2 HC and 10 PLWH with normal and low CD4/CD8 ratio post-vaccinations using virus neutralization test. No Nab titers were detected in pre-vaccination serum samples of all donors. Horizontal lines indicate median titer values; black circles indicate samples. Differences in Nab titers however were not significant among patient groups.

Supplementary Figure 4 Inter-individual variability in monocyte transcriptional responses following vaccination. Volcano plots showing differential gene expression in monocytes at the individual patient level (post- vs. pre-vaccination). Each panel represents a single donor, grouped by CD4/CD8 ratio category (healthy controls, normal, and low). Substantial inter-individual variability in gene-level responses is observed, with some individuals exhibiting strong transcriptional changes while others show more modest responses.

Supplementary Figure 5 Comparison of transcriptional complexity in monocytes across HC and PLWH groups. **(A)** Violin plots show the number of detected genes per monocyte (nFeature_RNA) across HC, PLWH with normal CD4/CD8 ratios, and PLWH with low CD4/CD8 ratios, pooled across timepoints. **(B)** Boxplots show the mean number of detected genes per monocyte, averaged at the individual level. Each point represents one individual. These analyses indicate that the increased node degree observed in monocyte PPI networks from PLWH cannot be attributed to higher transcriptional complexity.

Supplementary Figure 6 Differential gene expression in monocytes at the pre-vaccination timepoint. Volcano plots showing differential gene expression in monocytes at baseline (pre-vaccination) for comparisons between low CD4/CD8 ratio vs. healthy controls (top), normal CD4/CD8 ratio vs. healthy controls (middle), and low vs. normal CD4/CD8 ratio groups (bottom). Selected genes are annotated. While stress response–related transcripts (e.g., *JUNB*, *DUSP1*, *ANXA1*) were detectable in PLWH compared to healthy controls at baseline, these signatures were not specifically enriched in the low CD4/CD8 group relative to the normal CD4/CD8 group. Overall, similar expression patterns were observed across PLWH subgroups, indicating that the stress-related transcriptional features observed post-vaccination are not pre-existing characteristics unique to the low CD4/CD8 group but instead emerge following vaccination.

Supplementary Figure 7 Inter-individual variability in pathway-level responses in monocytes following vaccination. Gene set enrichment analysis (GSEA) was performed at the individual patient level (post- vs. pre-vaccination) using Wilcoxon AUC–ranked genes. Each point represents the normalized enrichment score (NES) for a single patient. Hallmark pathways associated with antiviral responses (interferon alpha response, interferon gamma response, and TNFα signaling via NFκB) are grouped into the antiviral panel. Positive NES values indicate enrichment post-vaccination, whereas negative NES values indicate enrichment pre-vaccination. Boxplots summarize the distribution of NES values across patients within each CD4/CD8 ratio group, with the center line indicating the median and boxes representing the interquartile range.

Supplementary Table 1 Clinical characteristics and immunological parameters of study participants.

Supplementary Table 2 List of all differentially expressed genes across major immune cell group, post-vaccination vs pre-vaccination samples of HC and PLWH with normal and low CD4/CD8 ratio.

Supplementary Table 3 Gene sets used for network classification and visualization.

Supplementary Table 4 Network topology metrics of STRING-derived protein–protein interaction networks.

Supplementary Table 5 Frequency and proportion of top 10 antigen species predicted to be specific to the TCR clonotypes.
