# Supplementary material for: CD4/CD8 ratio is associated with structural reorganization of vaccine-induced immune responses in people living with HIV
Source: Front Immunol. 2026 Jun 1;17:1821444. doi: 10.3389/fimmu.2026.1821444 (PMC13265532; doi:10.3389/fimmu.2026.1821444)
Supplement: Supplementary Figure 1 — Integration of in-house and publicly available healthy control datasets. UMAP visualization of integrated single-cell RNA-seq data from in-house healthy controls and a publicly available healthy control dataset (GSE171964). Cells are colored by dataset origin (In-house HC vs. GSE HC). Cells from both datasets co-localize across shared clusters without evident dataset-specific segregation, indicating comparable transcriptional profiles. Despite the smaller number of cells in the publicly available dataset, its distribution across major immune cell populations supports its inclusion in downstream analyses. [file Presentation1.pdf]

CD4/CD8 ratio is associated with structural reorganization of vaccine-induced immune responses in people living with HIV

SUPPLEMENTARY FIGURES

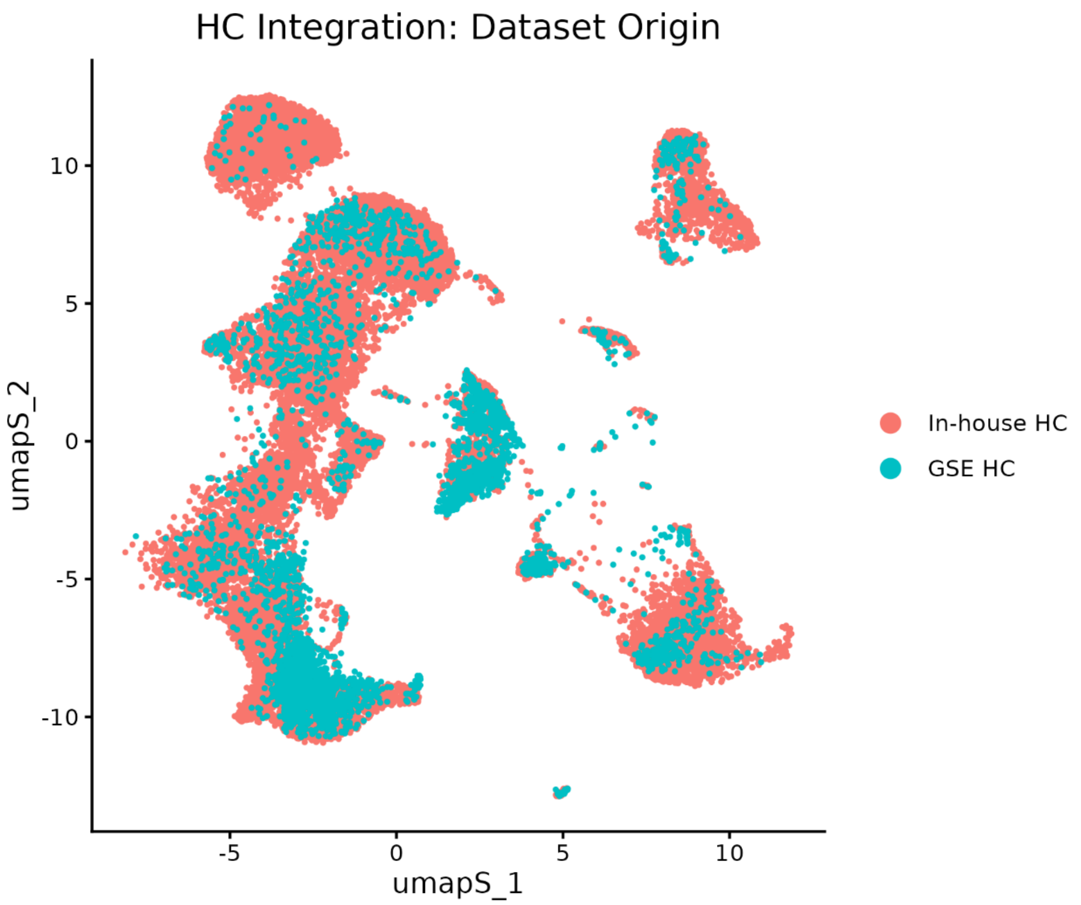

Supplementary Figure S1. Integration of in-house and publicly available healthy control datasets.

UMAP visualization of integrated single-cell RNA-seq data from in-house healthy controls and a publicly available healthy control dataset (GSE171964). Cells are colored by dataset origin (In-house HC vs. GSE HC). Cells from both datasets co-localize across shared clusters without evident dataset-specific segregation, indicating comparable transcriptional profiles. Despite the smaller number of cells in the publicly available dataset, its distribution across major immune cell populations supports its inclusion in downstream analyses.

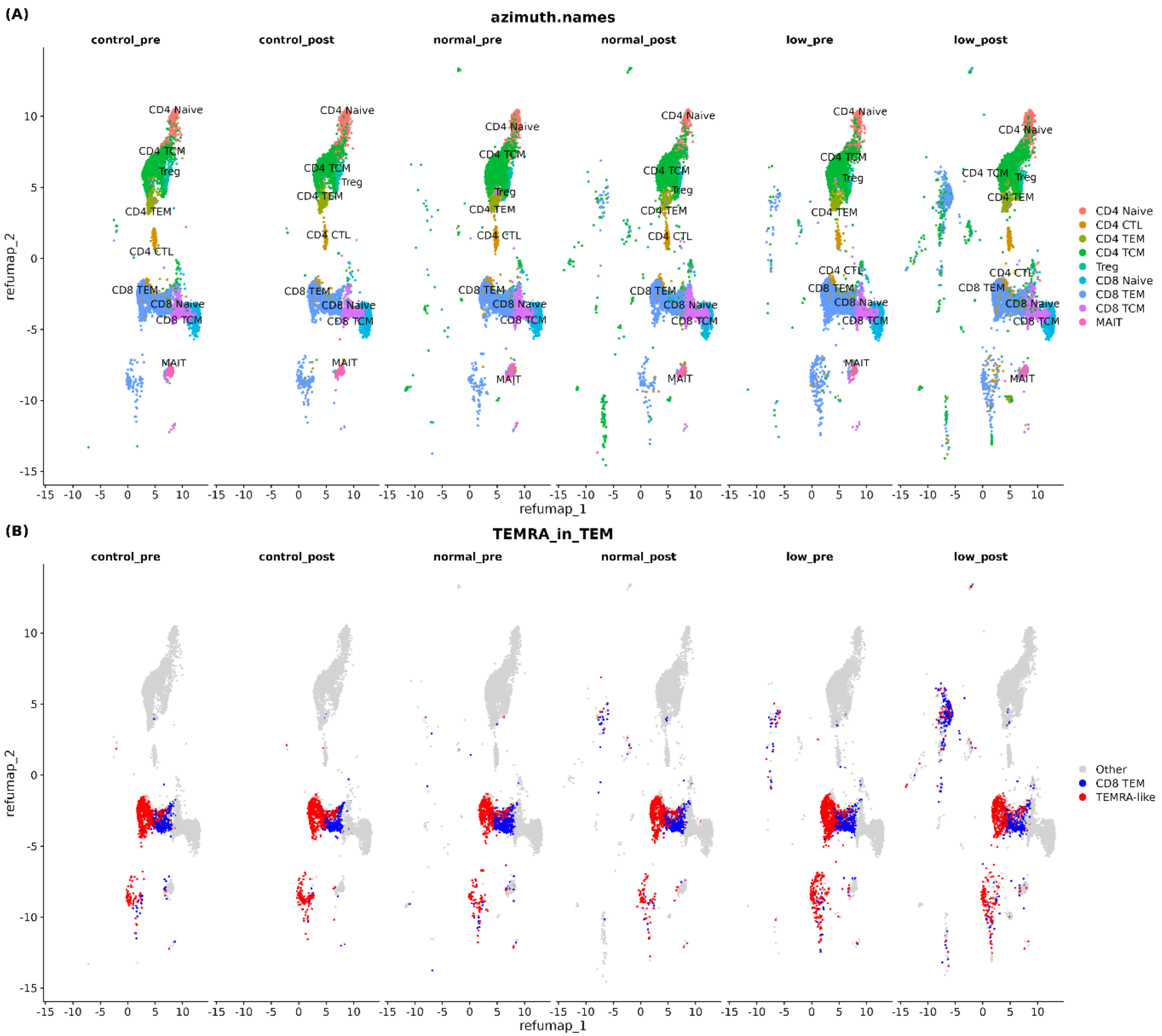

Supplementary Figure S2. Identification and distribution of TEMRA-like CD8⁺ T cells across cohorts and vaccination states. (A) UMAP projection of T cells colored by Azimuth-derived cell type annotations, split by cohort and vaccination status. Cell type labels are overlaid for reference. (B) UMAP highlighting TEMRA-like cells defined by a cytotoxicity and terminal differentiation gene module (*PTPRC, KLRG1, GNLY, GZMB, PRF1, FGFBP2*), as described in Methods. Cells exceeding the module score threshold were classified as "TEMRA-like," while remaining CD8 TEM cells and other populations are shown separately. TEMRA-like cells predominantly localize within the CD8 TEM compartment, consistent with the transcriptional continuum between effector memory and terminally differentiated cytotoxic CD8⁺ T cells.

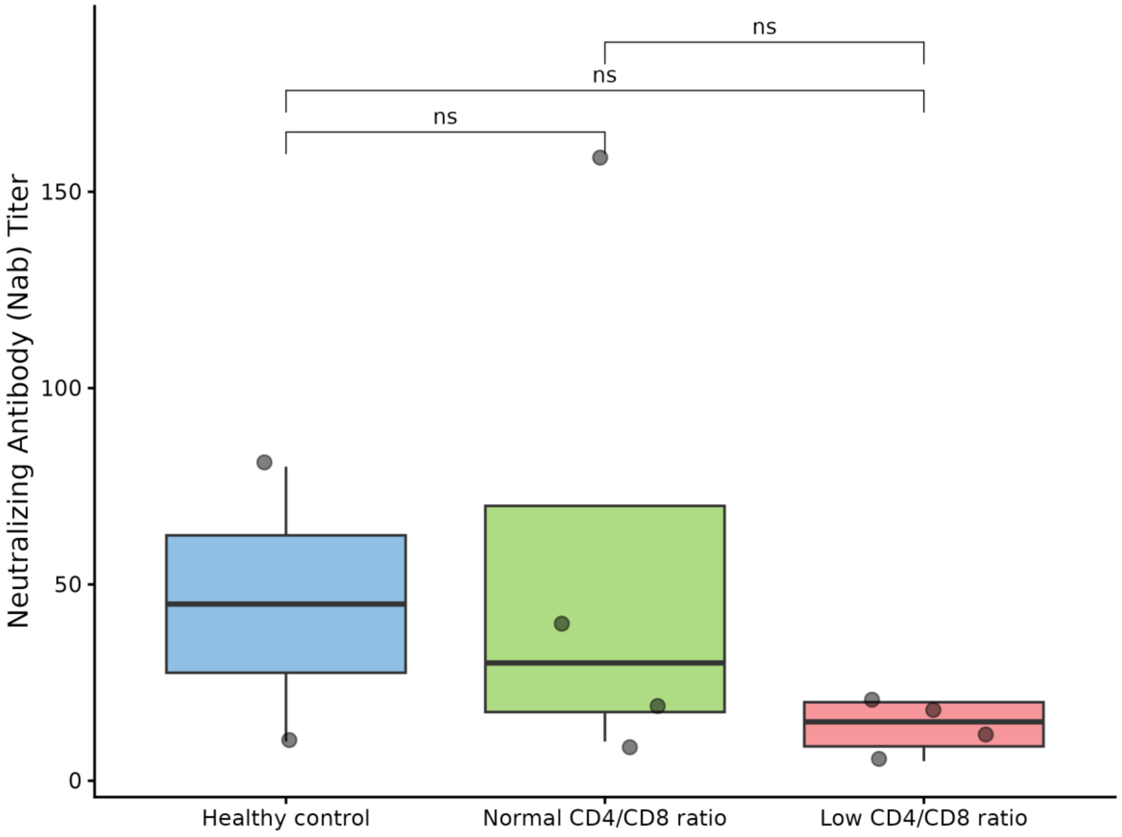

Supplementary Figure S3. Neutralizing antibody (Nab) titers among healthy control, normal CD4/CD8 ratio, and low CD4/CD8 ratio groups after COVID-19 vaccinations. Analysis of Nab titers against the SARS-CoV-2 Wuhan strain for serum samples from 2 HC and 10 PLWH with normal and low CD4/CD8 ratio post-vaccinations using virus neutralization test. No Nab titers were detected in pre-vaccination serum samples of all donors. Horizontal lines indicate median titer values; black circles indicate samples. Differences in Nab titers however were not significant among patient groups.

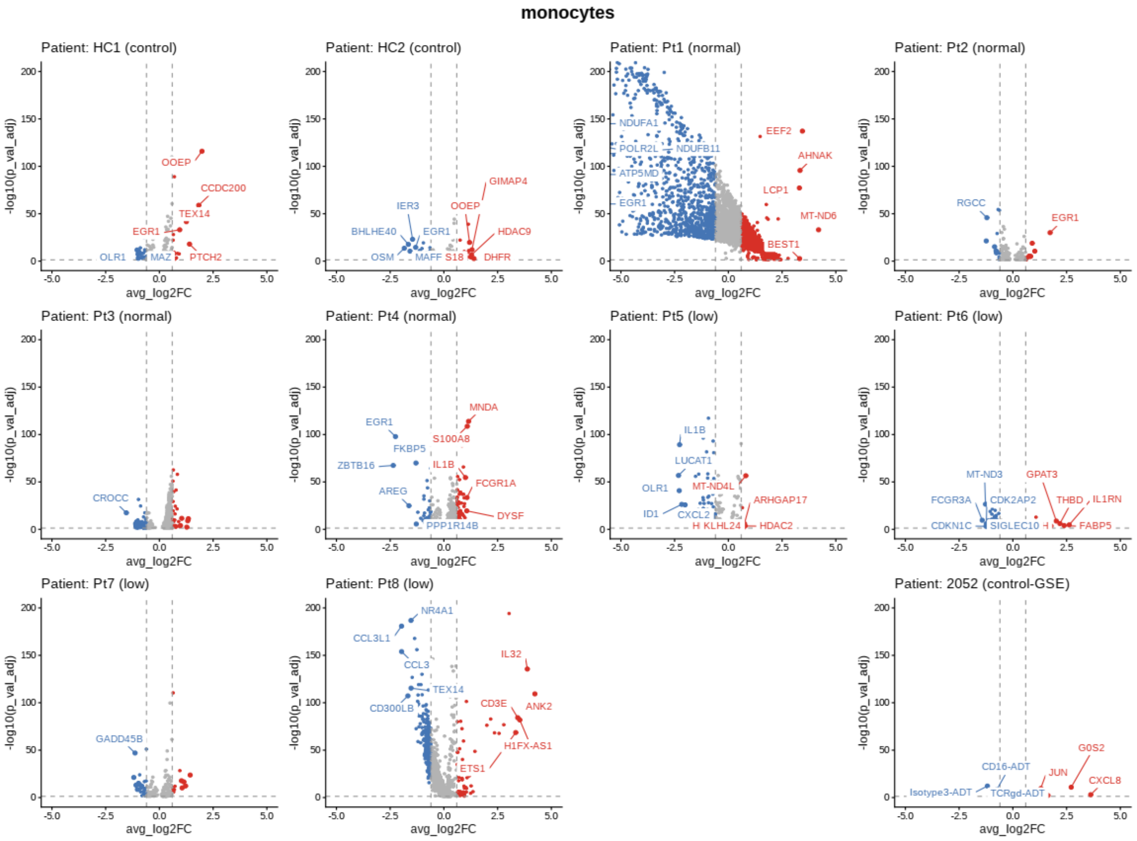

Supplementary Figure 4. Inter-individual variability in monocyte transcriptional responses following vaccination.

Volcano plots showing differential gene expression in monocytes at the individual patient level (post- vs. pre-vaccination). Each panel represents a single donor, grouped by CD4/CD8 ratio category (healthy controls, normal, and low). Substantial inter-individual variability in gene-level responses is observed, with some individuals exhibiting strong transcriptional changes while others show more modest responses.

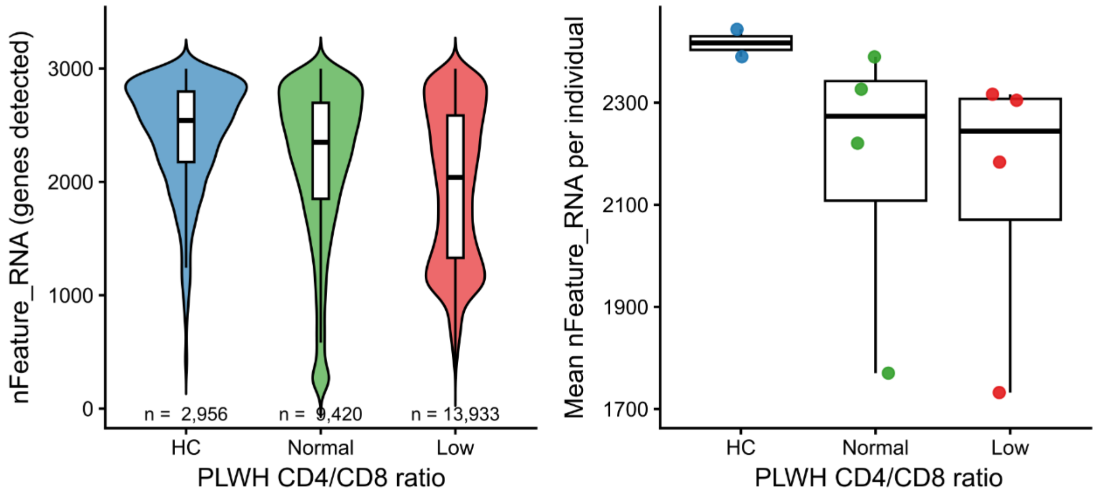

Supplementary Figure S5. Comparison of transcriptional complexity in monocytes across HC and PLWH groups. (A) Violin plots show the number of detected genes per monocyte (nFeature_RNA) across HC, PLWH with normal CD4/CD8 ratios, and PLWH with low CD4/CD8 ratios, pooled across timepoints. (B) Boxplots show the mean number of detected genes per monocyte, averaged at the individual level. Each point represents one individual. These analyses indicate that the increased node degree observed in monocyte PPI networks from PLWH cannot be attributed to higher transcriptional complexity.

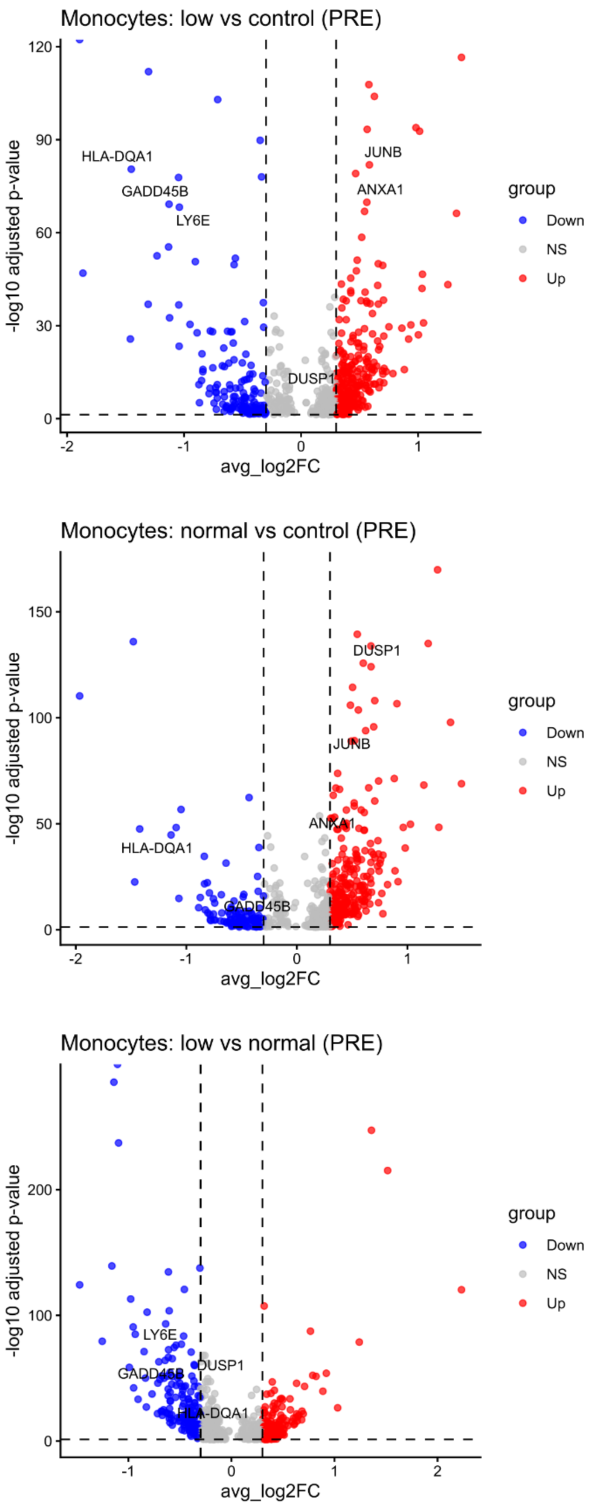

Supplementary Figure S6. Differential gene expression in monocytes at the pre-vaccination timepoint.

Volcano plots showing differential gene expression in monocytes at baseline (pre-vaccination) for comparisons between low CD4/CD8 ratio vs. healthy controls (top), normal CD4/CD8 ratio vs. healthy controls (middle), and low vs. normal CD4/CD8 ratio groups (bottom). Selected genes are annotated. While stress response–related transcripts (e.g., *JUNB*, *DUSP1*, *ANXA1*) were detectable in PLWH compared to healthy controls at baseline, these signatures were not specifically enriched in the low CD4/CD8 group relative to the normal CD4/CD8 group. Overall, similar expression patterns were observed across PLWH subgroups, indicating that the stress-related transcriptional features observed post-vaccination are not pre-existing characteristics unique to the low CD4/CD8 group but instead emerge following vaccination.

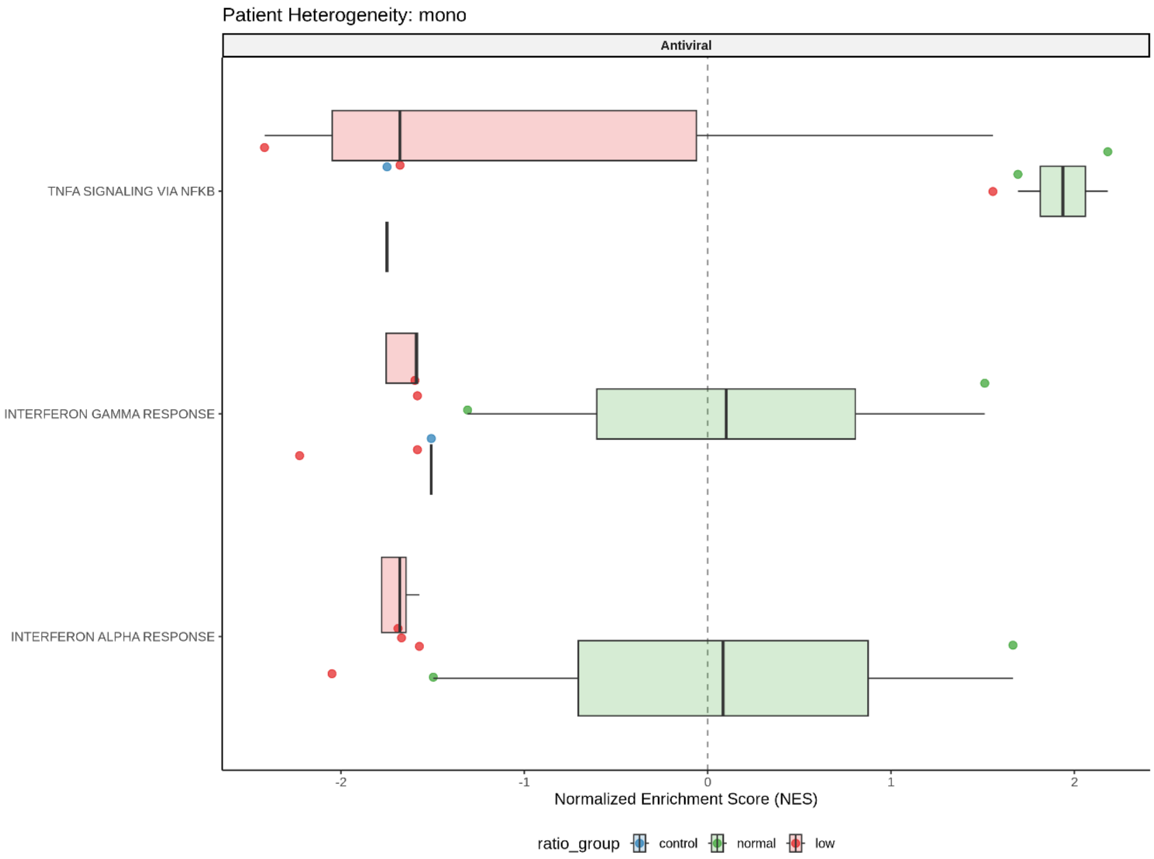

Supplementary Figure S7. Inter-individual variability in pathway-level responses in monocytes following vaccination. Gene set enrichment analysis (GSEA) was performed at the individual patient level (post- vs. pre-vaccination) using Wilcoxon AUC–ranked genes. Each point represents the normalized enrichment score (NES) for a single patient. Hallmark pathways associated with antiviral responses (interferon alpha response, interferon gamma response, and TNFα signaling via NFκB) are grouped into the antiviral panel. Positive NES values indicate enrichment post-vaccination, whereas negative NES values indicate enrichment pre-vaccination. Boxplots summarize the distribution of NES values across patients within each CD4/CD8 ratio group, with the center line indicating the median and boxes representing the interquartile range.
